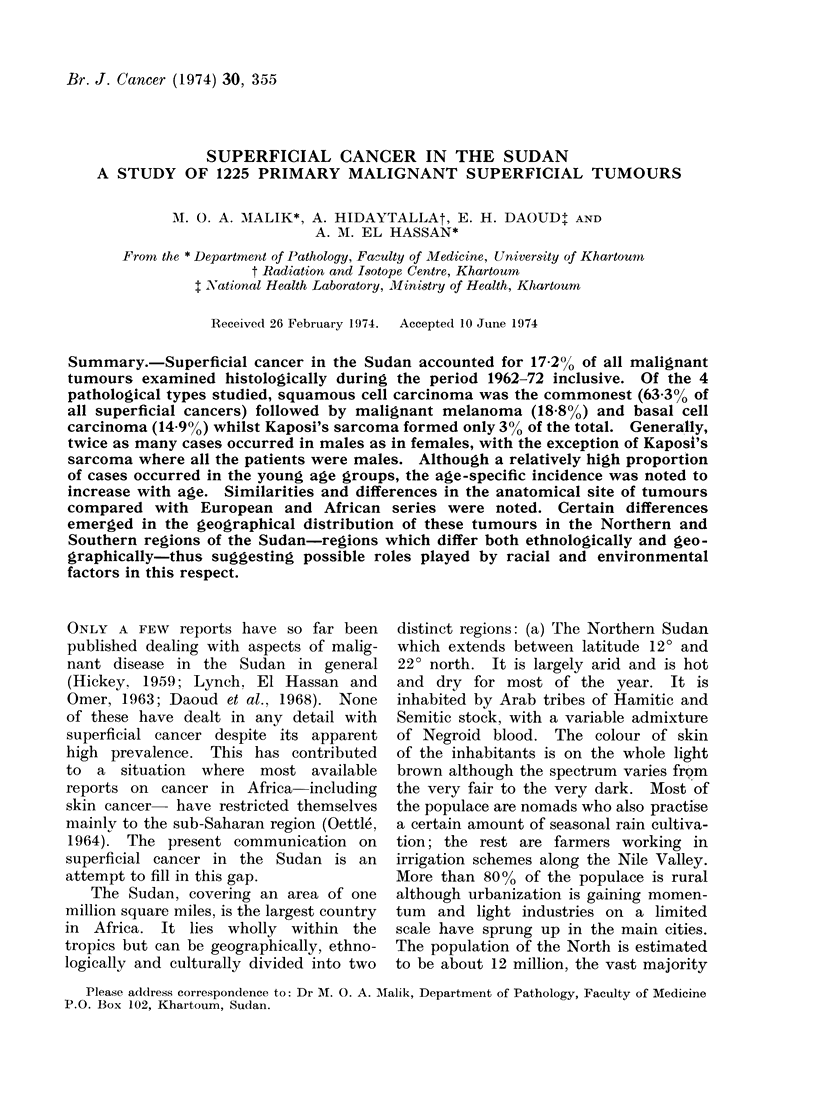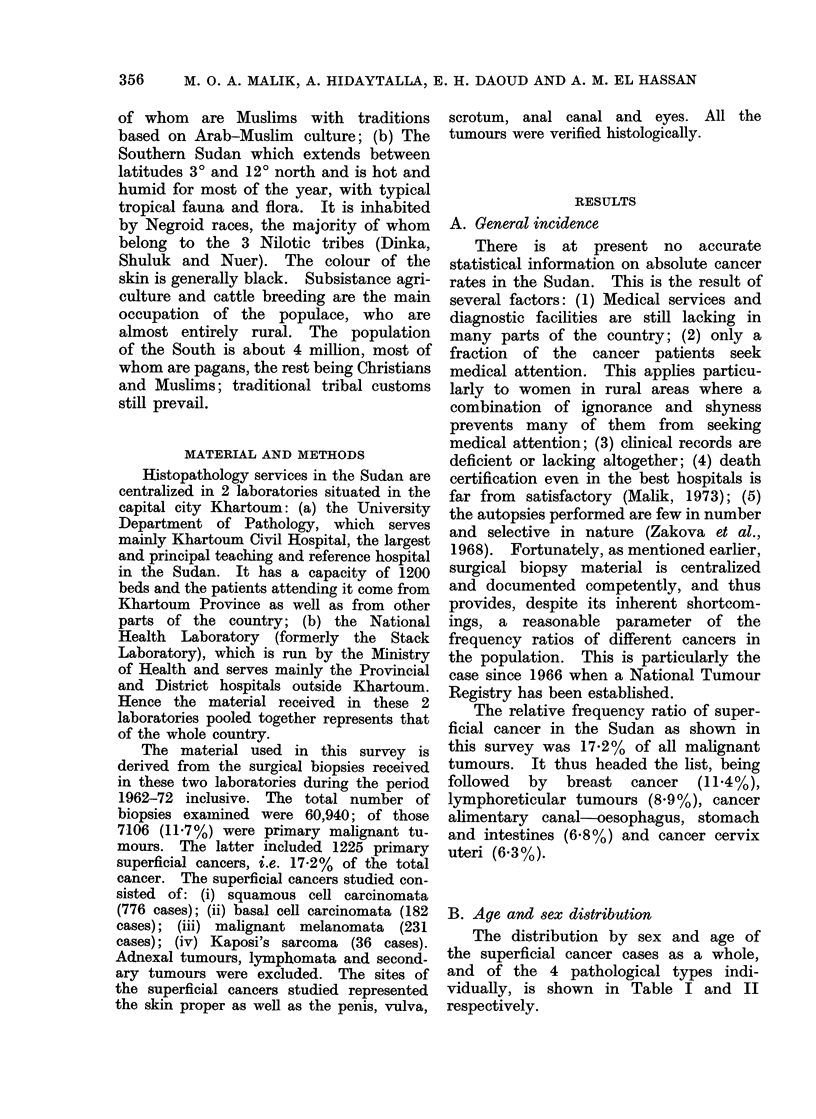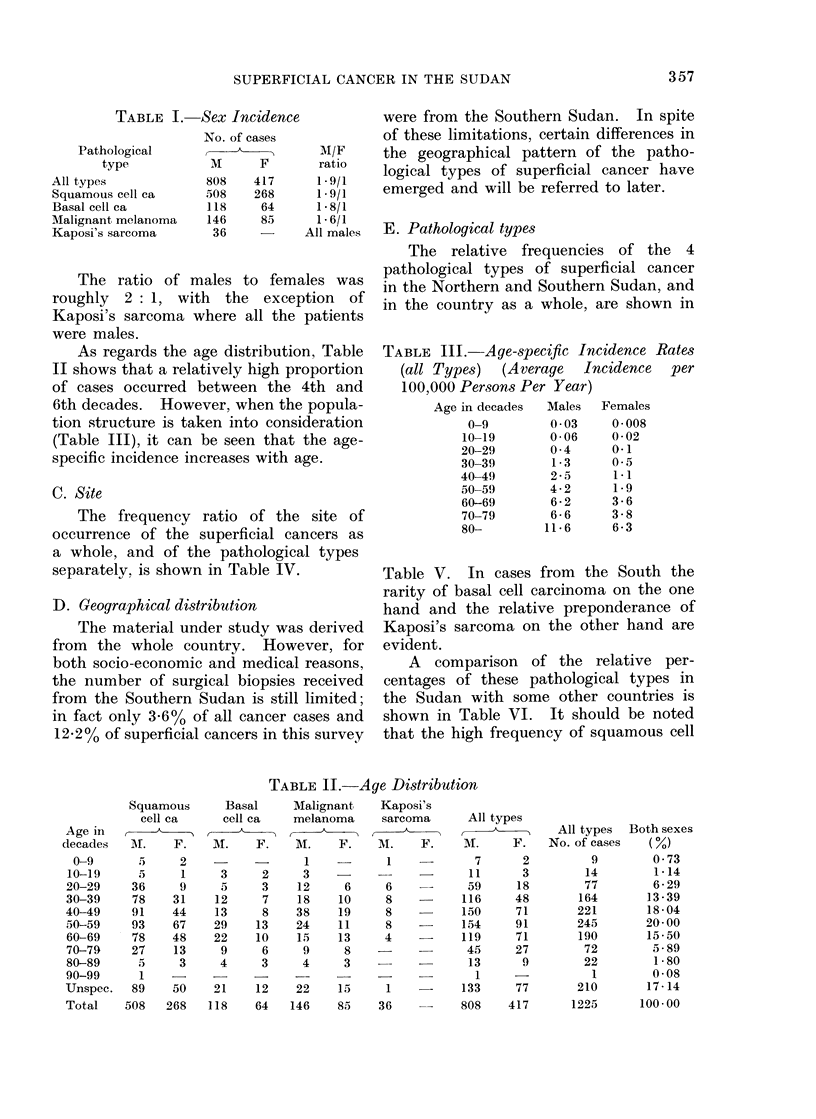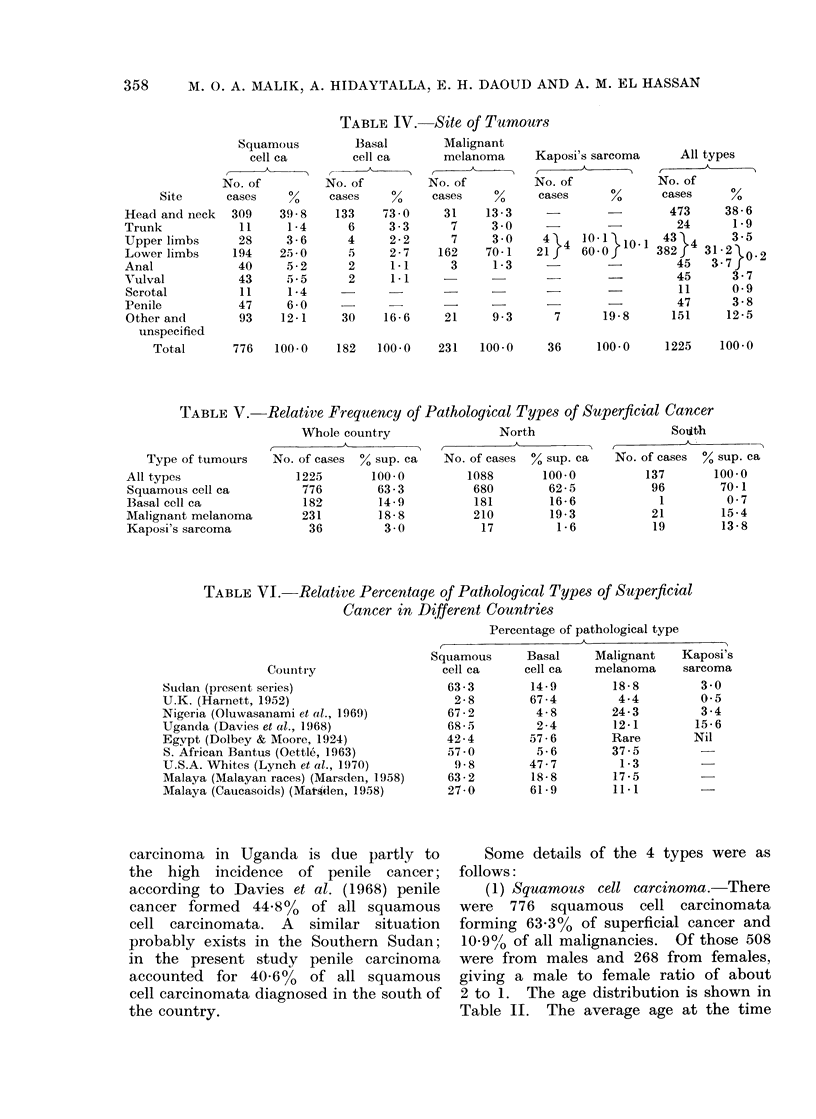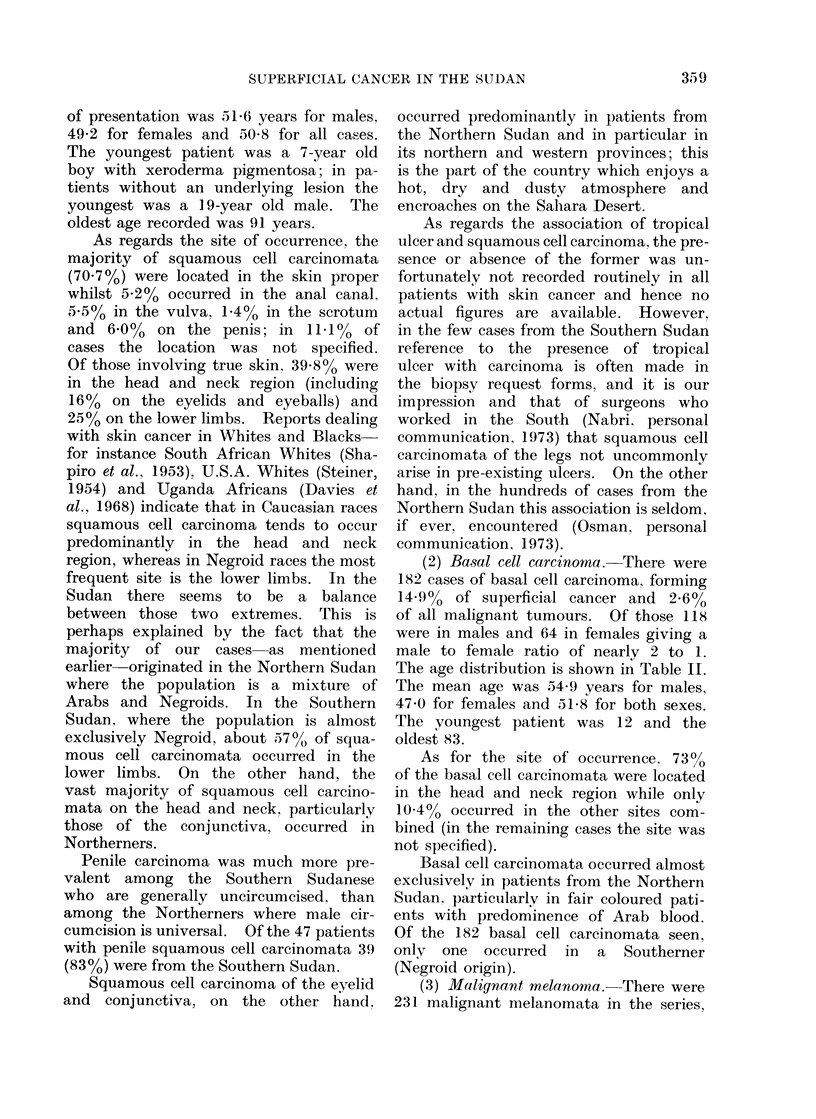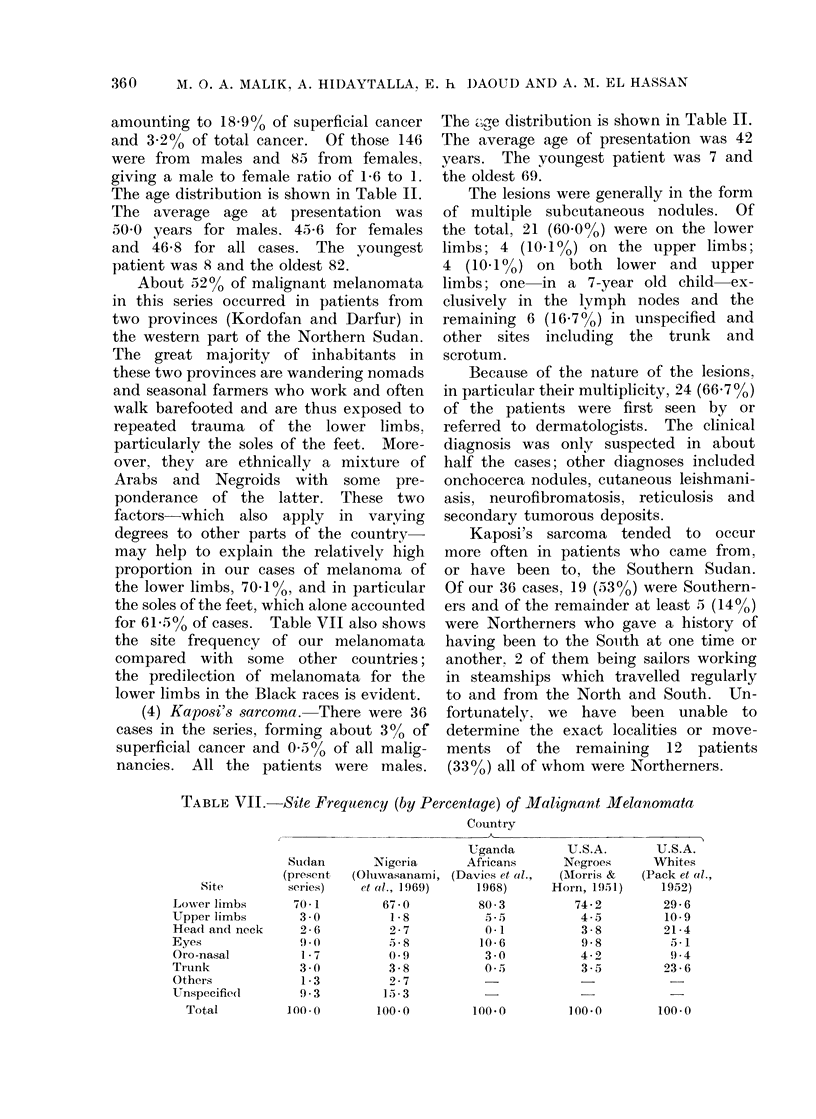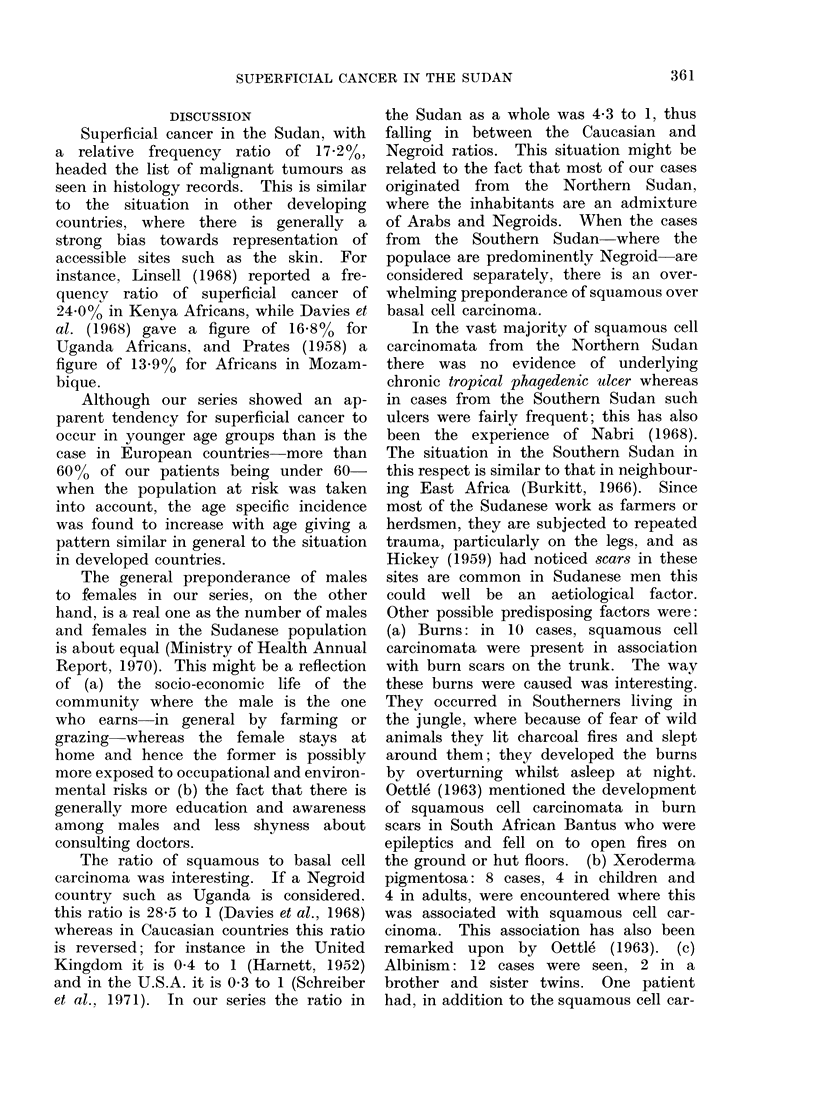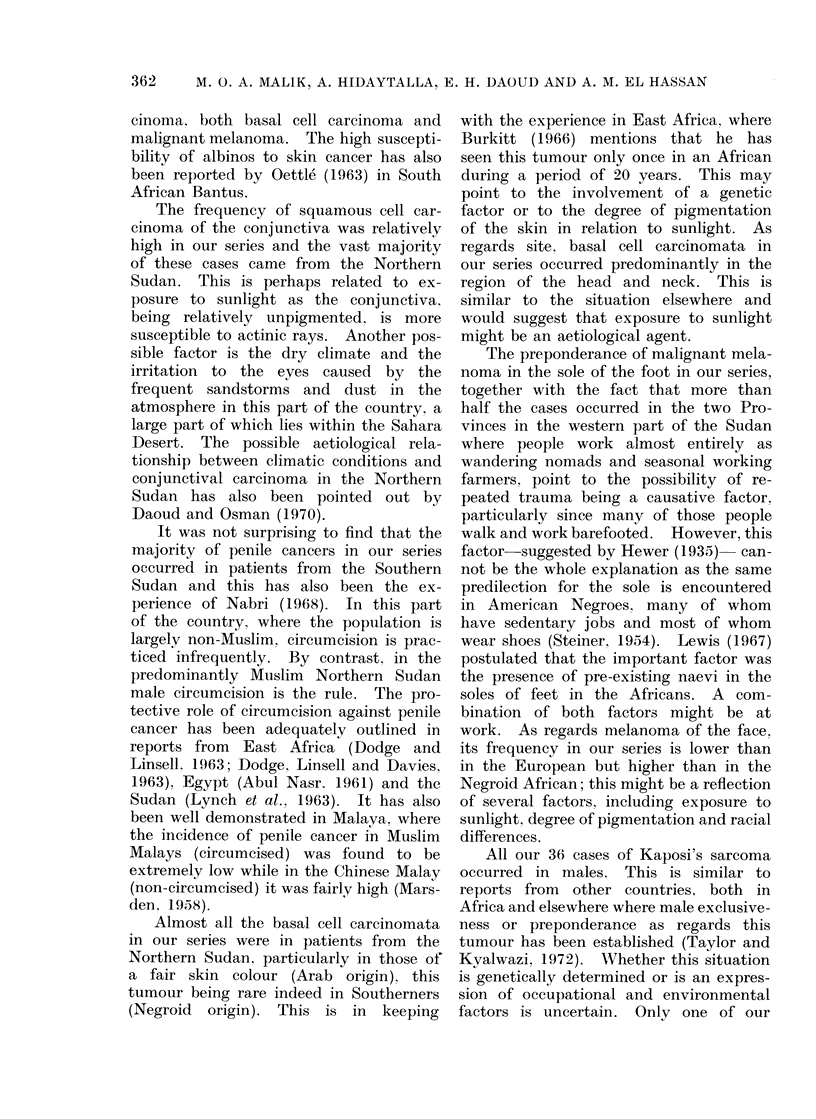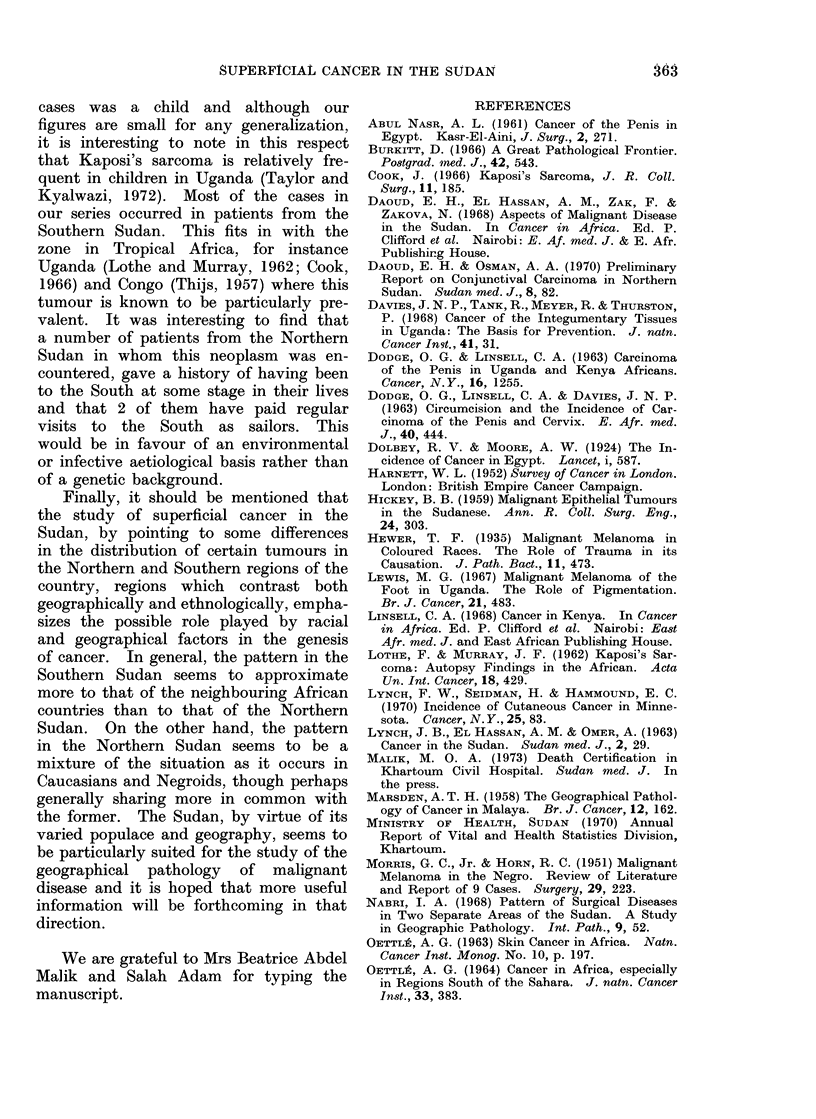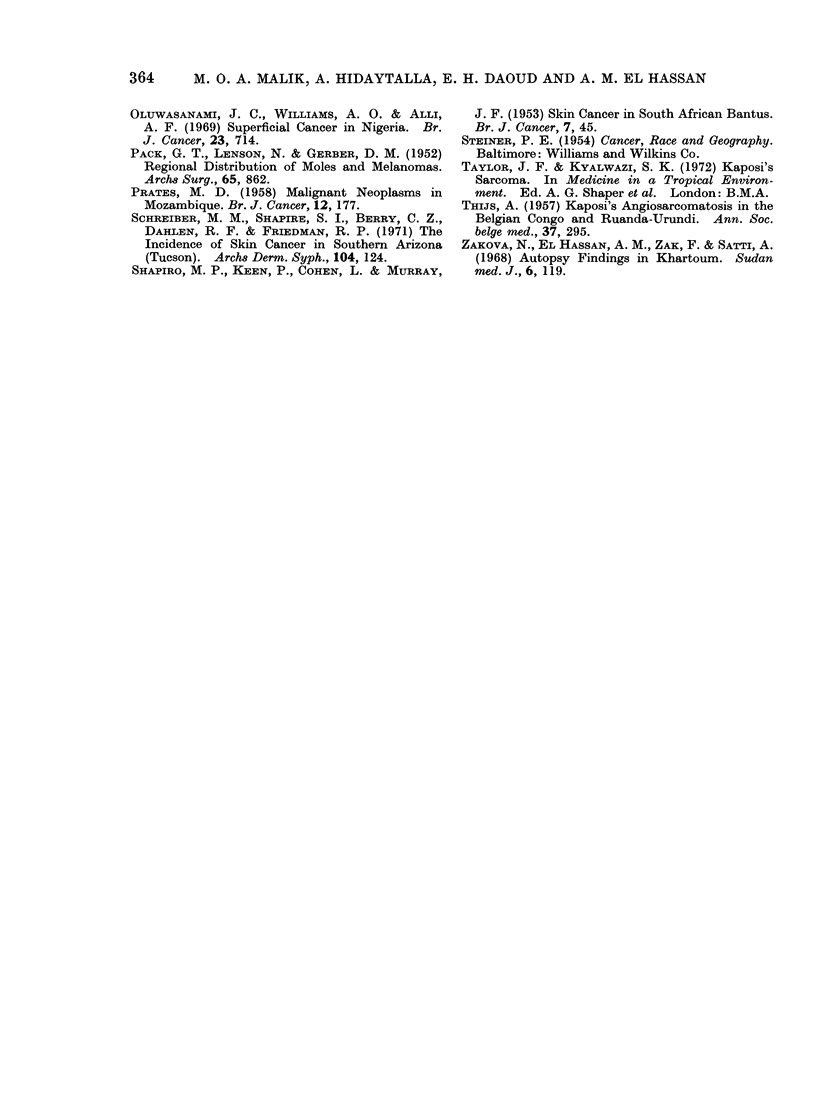# Superficial Cancer in the Sudan. A Study of 1225 Primary Malignant Superficial Tumours

**DOI:** 10.1038/bjc.1974.205

**Published:** 1974-10

**Authors:** M. O. A. Malik, A. Hidaytalla, E. H. Daoud, A. M. el Hassan

## Abstract

Superficial cancer in the Sudan accounted for 17·2% of all malignant tumours examined histologically during the period 1962-72 inclusive. Of the 4 pathological types studied, squamous cell carcinoma was the commonest (63·3% of all superficial cancers) followed by malignant melanoma (18·8%) and basal cell carcinoma (14·9%) whilst Kaposi's sarcoma formed only 3% of the total. Generally, twice as many cases occurred in males as in females, with the exception of Kaposi's sarcoma where all the patients were males. Although a relatively high proportion of cases occurred in the young age groups, the age-specific incidence was noted to increase with age. Similarities and differences in the anatomical site of tumours compared with European and African series were noted. Certain differences emerged in the geographical distribution of these tumours in the Northern and Southern regions of the Sudan—regions which differ both ethnologically and geographically—thus suggesting possible roles played by racial and environmental factors in this respect.


*Br. J. Cancer* (1974) **30**, 355

### SUPERFICIAL CANCER IN THE SUDAN
### A STUDY OF 1225 PRIMARY MALIGNANT SUPERFICIAL TUMOURS

M. O. A. MALIK*, A. HIDAYTALLA†, E. H. DAOUD‡ AND A. M. EL HASSAN*

*From the * Department of Pathology, Faculty of Medicine, University of Khartoum*
*† Radiation and Isotope Centre, Khartoum*
*‡ National Health Laboratory, Ministry of Health, Khartoum*

Received 26 February 1974. Accepted 10 June 1974

**Summary.—Superficial cancer in the Sudan accounted for 17·2% of all malignant tumours examined histologically during the period 1962–72 inclusive. Of the 4 pathological types studied, squamous cell carcinoma was the commonest (63·3% of all superficial cancers) followed by malignant melanoma (18·8%) and basal cell carcinoma (14·9%) whilst Kaposi's sarcoma formed only 3% of the total. Generally, twice as many cases occurred in males as in females, with the exception of Kaposi's sarcoma where all the patients were males. Although a relatively high proportion of cases occurred in the young age groups, the age-specific incidence was noted to increase with age. Similarities and differences in the anatomical site of tumours compared with European and African series were noted. Certain differences emerged in the geographical distribution of these tumours in the Northern and Southern regions of the Sudan—regions which differ both ethnologically and geographically—thus suggesting possible roles played by racial and environmental factors in this respect.**

ONLY A FEW reports have so far been published dealing with aspects of malignant disease in the Sudan in general (Hickey, 1959; Lynch, El Hassan and Omer, 1963; Daoud *et al.*, 1968). None of these have dealt in any detail with superficial cancer despite its apparent high prevalence. This has contributed to a situation where most available reports on cancer in Africa—including skin cancer— have restricted themselves mainly to the sub-Saharan region (Oettlé, 1964). The present communication on superficial cancer in the Sudan is an attempt to fill in this gap.

The Sudan, covering an area of one million square miles, is the largest country in Africa. It lies wholly within the tropics but can be geographically, ethnologically and culturally divided into two distinct regions: (a) The Northern Sudan which extends between latitude 12° and 22° north. It is largely arid and is hot and dry for most of the year. It is inhabited by Arab tribes of Hamitic and Semitic stock, with a variable admixture of Negroid blood. The colour of skin of the inhabitants is on the whole light brown although the spectrum varies from the very fair to the very dark. Most of the populace are nomads who also practise a certain amount of seasonal rain cultivation; the rest are farmers working in irrigation schemes along the Nile Valley. More than 80% of the populace is rural although urbanization is gaining momentum and light industries on a limited scale have sprung up in the main cities. The population of the North is estimated to be about 12 million, the vast majority

Please address correspondence to: Dr M. O. A. Malik, Department of Pathology, Faculty of Medicine P.O. Box 102, Khartoum, Sudan.

of whom are Muslims with traditions based on Arab–Muslim culture; (b) The Southern Sudan which extends between latitudes 3° and 12° north and is hot and humid for most of the year, with typical tropical fauna and flora. It is inhabited by Negroid races, the majority of whom belong to the 3 Nilotic tribes (Dinka, Shuluk and Nuer). The colour of the skin is generally black. Subsistance agriculture and cattle breeding are the main occupation of the populace, who are almost entirely rural. The population of the South is about 4 million, most of whom are pagans, the rest being Christians and Muslims; traditional tribal customs still prevail.

### MATERIAL AND METHODS

Histopathology services in the Sudan are centralized in 2 laboratories situated in the capital city Khartoum: (a) the University Department of Pathology, which serves mainly Khartoum Civil Hospital, the largest and principal teaching and reference hospital in the Sudan. It has a capacity of 1200 beds and the patients attending it come from Khartoum Province as well as from other parts of the country; (b) the National Health Laboratory (formerly the Stack Laboratory), which is run by the Ministry of Health and serves mainly the Provincial and District hospitals outside Khartoum. Hence the material received in these 2 laboratories pooled together represents that of the whole country.

The material used in this survey is derived from the surgical biopsies received in these two laboratories during the period 1962–72 inclusive. The total number of biopsies examined were 60,940; of those 7106 (11·7%) were primary malignant tumours. The latter included 1225 primary superficial cancers, *i.e.* 17·2% of the total cancer. The superficial cancers studied consisted of: (i) squamous cell carcinomata (776 cases); (ii) basal cell carcinomata (182 cases); (iii) malignant melanomata (231 cases); (iv) Kaposi's sarcoma (36 cases). Adnexal tumours, lymphomata and secondary tumours were excluded. The sites of the superficial cancers studied represented the skin proper as well as the penis, vulva, scrotum, anal canal and eyes. All the tumours were verified histologically.

### RESULTS

### A. *General incidence*

There is at present no accurate statistical information on absolute cancer rates in the Sudan. This is the result of several factors: (1) Medical services and diagnostic facilities are still lacking in many parts of the country; (2) only a fraction of the cancer patients seek medical attention. This applies particularly to women in rural areas where a combination of ignorance and shyness prevents many of them from seeking medical attention; (3) clinical records are deficient or lacking altogether; (4) death certification even in the best hospitals is far from satisfactory (Malik, 1973); (5) the autopsies performed are few in number and selective in nature (Zakova *et al.*, 1968). Fortunately, as mentioned earlier, surgical biopsy material is centralized and documented competently, and thus provides, despite its inherent shortcomings, a reasonable parameter of the frequency ratios of different cancers in the population. This is particularly the case since 1966 when a National Tumour Registry has been established.

The relative frequency ratio of superficial cancer in the Sudan as shown in this survey was 17·2% of all malignant tumours. It thus headed the list, being followed by breast cancer (11·4%), lymphoreticular tumours (8·9%), cancer alimentary canal—oesophagus, stomach and intestines (6·8%) and cancer cervix uteri (6·3%).

### B. *Age and sex distribution*

The distribution by sex and age of the superficial cancer cases as a whole, and of the 4 pathological types individually, is shown in Table I and II respectively.

TABLE I.—*Sex Incidence*

| Pathological type | No. of cases M | F | M/F ratio |
|---|---|---|---|
| All types | 808 | 417 | 1·9/1 |
| Squamous cell ca | 508 | 268 | 1·9/1 |
| Basal cell ca | 118 | 64 | 1·8/1 |
| Malignant melanoma | 146 | 85 | 1·6/1 |
| Kaposi's sarcoma | 36 | — | All males |

The ratio of males to females was roughly 2 : 1, with the exception of Kaposi's sarcoma where all the patients were males.

As regards the age distribution, Table II shows that a relatively high proportion of cases occurred between the 4th and 6th decades. However, when the population structure is taken into consideration (Table III), it can be seen that the age-specific incidence increases with age.

C. *Site*

The frequency ratio of the site of occurrence of the superficial cancers as a whole, and of the pathological types separately, is shown in Table IV.

D. *Geographical distribution*

The material under study was derived from the whole country. However, for both socio-economic and medical reasons, the number of surgical biopsies received from the Southern Sudan is still limited; in fact only 3·6% of all cancer cases and 12·2% of superficial cancers in this survey were from the Southern Sudan. In spite of these limitations, certain differences in the geographical pattern of the pathological types of superficial cancer have emerged and will be referred to later.

E. *Pathological types*

The relative frequencies of the 4 pathological types of superficial cancer in the Northern and Southern Sudan, and in the country as a whole, are shown in Table V. In cases from the South the rarity of basal cell carcinoma on the one hand and the relative preponderance of Kaposi's sarcoma on the other hand are evident.

TABLE III.—*Age-specific Incidence Rates (all Types) (Average Incidence per* 100,000 *Persons Per Year)*

| Age in decades | Males | Females |
|---|---|---|
| 0–9 | 0·03 | 0·008 |
| 10–19 | 0·06 | 0·02 |
| 20–29 | 0·4 | 0·1 |
| 30–39 | 1·3 | 0·5 |
| 40–49 | 2·5 | 1·1 |
| 50–59 | 4·2 | 1·9 |
| 60–69 | 6·2 | 3·6 |
| 70–79 | 6·6 | 3·8 |
| 80– | 11·6 | 6·3 |

A comparison of the relative percentages of these pathological types in the Sudan with some other countries is shown in Table VI. It should be noted that the high frequency of squamous cell

TABLE II.—*Age Distribution*

| Age in decades | Squamous cell ca M. | F. | Basal cell ca M. | F. | Malignant melanoma M. | F. | Kaposi's sarcoma M. | F. | All types M. | F. | All types No. of cases | Both sexes (%) |
|---|---|---|---|---|---|---|---|---|---|---|---|---|
| 0–9 | 5 | 2 | — | — | 1 | — | 1 | — | 7 | 2 | 9 | 0·73 |
| 10–19 | 5 | 1 | 3 | 2 | 3 | — | — | — | 11 | 3 | 14 | 1·14 |
| 20–29 | 36 | 9 | 5 | 3 | 12 | 6 | 6 | — | 59 | 18 | 77 | 6·29 |
| 30–39 | 78 | 31 | 12 | 7 | 18 | 10 | 8 | — | 116 | 48 | 164 | 13·39 |
| 40–49 | 91 | 44 | 13 | 8 | 38 | 19 | 8 | — | 150 | 71 | 221 | 18·04 |
| 50–59 | 93 | 67 | 29 | 13 | 24 | 11 | 8 | — | 154 | 91 | 245 | 20·00 |
| 60–69 | 78 | 48 | 22 | 10 | 15 | 13 | 4 | — | 119 | 71 | 190 | 15·50 |
| 70–79 | 27 | 13 | 9 | 6 | 9 | 8 | — | — | 45 | 27 | 72 | 5·89 |
| 80–89 | 5 | 3 | 4 | 3 | 4 | 3 | — | — | 13 | 9 | 22 | 1·80 |
| 90–99 | 1 | — | — | — | — | — | — | — | 1 | — | 1 | 0·08 |
| Unspec. | 89 | 50 | 21 | 12 | 22 | 15 | 1 | — | 133 | 77 | 210 | 17·14 |
| Total | 508 | 268 | 118 | 64 | 146 | 85 | 36 | — | 808 | 417 | 1225 | 100·00 |

TABLE IV.—*Site of Tumours*

| Site | Squamous cell ca | | Basal cell ca | | Malignant melanoma | | Kaposi's sarcoma | | All types | |
|---|---|---|---|---|---|---|---|---|---|---|
| | No. of cases | % | No. of cases | % | No. of cases | % | No. of cases | % | No. of cases | % |
| Head and neck | 309 | 39·8 | 133 | 73·0 | 31 | 13·3 | — | — | 473 | 38·6 |
| Trunk | 11 | 1·4 | 6 | 3·3 | 7 | 3·0 | — | — | 24 | 1·9 |
| Upper limbs | 28 | 3·6 | 4 | 2·2 | 7 | 3·0 | 4 }4 | 10·1 }10·1 | 43 }4 | 3·5 |
| Lower limbs | 194 | 25·0 | 5 | 2·7 | 162 | 70·1 | 21 } | 60·0 } | 382 } | 31·2 }0·2 |
| Anal | 40 | 5·2 | 2 | 1·1 | 3 | 1·3 | — | — | 45 | 3·7 } |
| Vulval | 43 | 5·5 | 2 | 1·1 | — | — | — | — | 45 | 3·7 |
| Scrotal | 11 | 1·4 | — | — | — | — | — | — | 11 | 0·9 |
| Penile | 47 | 6·0 | — | — | — | — | — | — | 47 | 3·8 |
| Other and unspecified | 93 | 12·1 | 30 | 16·6 | 21 | 9·3 | 7 | 19·8 | 151 | 12·5 |
| Total | 776 | 100·0 | 182 | 100·0 | 231 | 100·0 | 36 | 100·0 | 1225 | 100·0 |

TABLE V.—*Relative Frequency of Pathological Types of Superficial Cancer*

| Type of tumours | Whole country | | North | | South | |
|---|---|---|---|---|---|---|
| | No. of cases | % sup. ca | No. of cases | % sup. ca | No. of cases | % sup. ca |
| All types | 1225 | 100·0 | 1088 | 100·0 | 137 | 100·0 |
| Squamous cell ca | 776 | 63·3 | 680 | 62·5 | 96 | 70·1 |
| Basal cell ca | 182 | 14·9 | 181 | 16·6 | 1 | 0·7 |
| Malignant melanoma | 231 | 18·8 | 210 | 19·3 | 21 | 15·4 |
| Kaposi's sarcoma | 36 | 3·0 | 17 | 1·6 | 19 | 13·8 |

TABLE VI.—*Relative Percentage of Pathological Types of Superficial Cancer in Different Countries*

| Country | Percentage of pathological type | | | |
|---|---|---|---|---|
| | Squamous cell ca | Basal cell ca | Malignant melanoma | Kaposi's sarcoma |
| Sudan (present series) | 63·3 | 14·9 | 18·8 | 3·0 |
| U.K. (Harnett, 1952) | 2·8 | 67·4 | 4·4 | 0·5 |
| Nigeria (Oluwasanami *et al.*, 1969) | 67·2 | 4·8 | 24·3 | 3·4 |
| Uganda (Davies *et al.*, 1968) | 68·5 | 2·4 | 12·1 | 15·6 |
| Egypt (Dolbey & Moore, 1924) | 42·4 | 57·6 | Rare | Nil |
| S. African Bantus (Oettlé, 1963) | 57·0 | 5·6 | 37·5 | — |
| U.S.A. Whites (Lynch *et al.*, 1970) | 9·8 | 47·7 | 1·3 | — |
| Malaya (Malayan races) (Marsden, 1958) | 63·2 | 18·8 | 17·5 | — |
| Malaya (Caucasoids) (Marsden, 1958) | 27·0 | 61·9 | 11·1 | — |

carcinoma in Uganda is due partly to the high incidence of penile cancer; according to Davies *et al.* (1968) penile cancer formed 44·8% of all squamous cell carcinomata. A similar situation probably exists in the Southern Sudan; in the present study penile carcinoma accounted for 40·6% of all squamous cell carcinomata diagnosed in the south of the country.

Some details of the 4 types were as follows:

(1) *Squamous cell carcinoma.*—There were 776 squamous cell carcinomata forming 63·3% of superficial cancer and 10·9% of all malignancies. Of those 508 were from males and 268 from females, giving a male to female ratio of about 2 to 1. The age distribution is shown in Table II. The average age at the time of presentation was 51·6 years for males, 49·2 for females and 50·8 for all cases. The youngest patient was a 7-year old boy with xeroderma pigmentosa; in patients without an underlying lesion the youngest was a 19-year old male. The oldest age recorded was 91 years.

As regards the site of occurrence, the majority of squamous cell carcinomata (70·7%) were located in the skin proper whilst 5·2% occurred in the anal canal, 5·5% in the vulva, 1·4% in the scrotum and 6·0% on the penis; in 11·1% of cases the location was not specified. Of those involving true skin, 39·8% were in the head and neck region (including 16% on the eyelids and eyeballs) and 25% on the lower limbs. Reports dealing with skin cancer in Whites and Blacks—for instance South African Whites (Shapiro *et al.*, 1953), U.S.A. Whites (Steiner, 1954) and Uganda Africans (Davies *et al.*, 1968) indicate that in Caucasian races squamous cell carcinoma tends to occur predominantly in the head and neck region, whereas in Negroid races the most frequent site is the lower limbs. In the Sudan there seems to be a balance between those two extremes. This is perhaps explained by the fact that the majority of our cases—as mentioned earlier—originated in the Northern Sudan where the population is a mixture of Arabs and Negroids. In the Southern Sudan, where the population is almost exclusively Negroid, about 57% of squamous cell carcinomata occurred in the lower limbs. On the other hand, the vast majority of squamous cell carcinomata on the head and neck, particularly those of the conjunctiva, occurred in Northerners.

Penile carcinoma was much more prevalent among the Southern Sudanese who are generally uncircumcised, than among the Northerners where male circumcision is universal. Of the 47 patients with penile squamous cell carcinomata 39 (83%) were from the Southern Sudan.

Squamous cell carcinoma of the eyelid and conjunctiva, on the other hand, occurred predominantly in patients from the Northern Sudan and in particular in its northern and western provinces; this is the part of the country which enjoys a hot, dry and dusty atmosphere and encroaches on the Sahara Desert.

As regards the association of tropical ulcer and squamous cell carcinoma, the presence or absence of the former was unfortunately not recorded routinely in all patients with skin cancer and hence no actual figures are available. However, in the few cases from the Southern Sudan reference to the presence of tropical ulcer with carcinoma is often made in the biopsy request forms, and it is our impression and that of surgeons who worked in the South (Nabri, personal communication, 1973) that squamous cell carcinomata of the legs not uncommonly arise in pre-existing ulcers. On the other hand, in the hundreds of cases from the Northern Sudan this association is seldom, if ever, encountered (Osman, personal communication, 1973).

(2) *Basal cell carcinoma.*—There were 182 cases of basal cell carcinoma, forming 14·9% of superficial cancer and 2·6% of all malignant tumours. Of those 118 were in males and 64 in females giving a male to female ratio of nearly 2 to 1. The age distribution is shown in Table II. The mean age was 54·9 years for males, 47·0 for females and 51·8 for both sexes. The youngest patient was 12 and the oldest 83.

As for the site of occurrence, 73% of the basal cell carcinomata were located in the head and neck region while only 10·4% occurred in the other sites combined (in the remaining cases the site was not specified).

Basal cell carcinomata occurred almost exclusively in patients from the Northern Sudan, particularly in fair coloured patients with predominence of Arab blood. Of the 182 basal cell carcinomata seen, only one occurred in a Southerner (Negroid origin).

(3) *Malignant melanoma.*—There were 231 malignant melanomata in the series, amounting to 18·9% of superficial cancer and 3·2% of total cancer. Of those 146 were from males and 85 from females, giving a male to female ratio of 1·6 to 1. The age distribution is shown in Table II. The average age at presentation was 50·0 years for males, 45·6 for females and 46·8 for all cases. The youngest patient was 8 and the oldest 82.

About 52% of malignant melanomata in this series occurred in patients from two provinces (Kordofan and Darfur) in the western part of the Northern Sudan. The great majority of inhabitants in these two provinces are wandering nomads and seasonal farmers who work and often walk barefooted and are thus exposed to repeated trauma of the lower limbs, particularly the soles of the feet. Moreover, they are ethnically a mixture of Arabs and Negroids with some preponderance of the latter. These two factors—which also apply in varying degrees to other parts of the country—may help to explain the relatively high proportion in our cases of melanoma of the lower limbs, 70·1%, and in particular the soles of the feet, which alone accounted for 61·5% of cases. Table VII also shows the site frequency of our melanomata compared with some other countries; the predilection of melanomata for the lower limbs in the Black races is evident.

(4) *Kaposi's sarcoma.*—There were 36 cases in the series, forming about 3% of superficial cancer and 0·5% of all malignancies. All the patients were males. The age distribution is shown in Table II. The average age of presentation was 42 years. The youngest patient was 7 and the oldest 69.

The lesions were generally in the form of multiple subcutaneous nodules. Of the total, 21 (60·0%) were on the lower limbs; 4 (10·1%) on the upper limbs; 4 (10·1%) on both lower and upper limbs; one—in a 7-year old child—exclusively in the lymph nodes and the remaining 6 (16·7%) in unspecified and other sites including the trunk and scrotum.

Because of the nature of the lesions, in particular their multiplicity, 24 (66·7%) of the patients were first seen by or referred to dermatologists. The clinical diagnosis was only suspected in about half the cases; other diagnoses included onchocerca nodules, cutaneous leishmaniasis, neurofibromatosis, reticulosis and secondary tumorous deposits.

Kaposi's sarcoma tended to occur more often in patients who came from, or have been to, the Southern Sudan. Of our 36 cases, 19 (53%) were Southerners and of the remainder at least 5 (14%) were Northerners who gave a history of having been to the South at one time or another, 2 of them being sailors working in steamships which travelled regularly to and from the North and South. Unfortunately, we have been unable to determine the exact localities or movements of the remaining 12 patients (33%) all of whom were Northerners.

TABLE VII.—*Site Frequency (by Percentage) of Malignant Melanomata*

| | Country | | | | |
|---|---|---|---|---|---|
| Site | Sudan (present series) | Nigeria (Oluwasanami, *et al.*, 1969) | Uganda Africans (Davies *et al.*, 1968) | U.S.A. Negroes (Morris & Horn, 1951) | U.S.A. Whites (Pack *et al.*, 1952) |
| Lower limbs | 70·1 | 67·0 | 80·3 | 74·2 | 29·6 |
| Upper limbs | 3·0 | 1·8 | 5·5 | 4·5 | 10·9 |
| Head and neck | 2·6 | 2·7 | 0·1 | 3·8 | 21·4 |
| Eyes | 9·0 | 5·8 | 10·6 | 9·8 | 5·1 |
| Oro-nasal | 1·7 | 0·9 | 3·0 | 4·2 | 9·4 |
| Trunk | 3·0 | 3·8 | 0·5 | 3·5 | 23·6 |
| Others | 1·3 | 2·7 | — | — | — |
| Unspecified | 9·3 | 15·3 | — | — | — |
| Total | 100·0 | 100·0 | 100·0 | 100·0 | 100·0 |

### DISCUSSION

Superficial cancer in the Sudan, with a relative frequency ratio of 17·2%, headed the list of malignant tumours as seen in histology records. This is similar to the situation in other developing countries, where there is generally a strong bias towards representation of accessible sites such as the skin. For instance, Linsell (1968) reported a frequency ratio of superficial cancer of 24·0% in Kenya Africans, while Davies *et al.* (1968) gave a figure of 16·8% for Uganda Africans, and Prates (1958) a figure of 13·9% for Africans in Mozambique.

Although our series showed an apparent tendency for superficial cancer to occur in younger age groups than is the case in European countries—more than 60% of our patients being under 60—when the population at risk was taken into account, the age specific incidence was found to increase with age giving a pattern similar in general to the situation in developed countries.

The general preponderance of males to females in our series, on the other hand, is a real one as the number of males and females in the Sudanese population is about equal (Ministry of Health Annual Report, 1970). This might be a reflection of (a) the socio-economic life of the community where the male is the one who earns—in general by farming or grazing—whereas the female stays at home and hence the former is possibly more exposed to occupational and environmental risks or (b) the fact that there is generally more education and awareness among males and less shyness about consulting doctors.

The ratio of squamous to basal cell carcinoma was interesting. If a Negroid country such as Uganda is considered, this ratio is 28·5 to 1 (Davies *et al.*, 1968) whereas in Caucasian countries this ratio is reversed; for instance in the United Kingdom it is 0·4 to 1 (Harnett, 1952) and in the U.S.A. it is 0·3 to 1 (Schreiber *et al.*, 1971). In our series the ratio in the Sudan as a whole was 4·3 to 1, thus falling in between the Caucasian and Negroid ratios. This situation might be related to the fact that most of our cases originated from the Northern Sudan, where the inhabitants are an admixture of Arabs and Negroids. When the cases from the Southern Sudan—where the populace are predominently Negroid—are considered separately, there is an overwhelming preponderance of squamous over basal cell carcinoma.

In the vast majority of squamous cell carcinomata from the Northern Sudan there was no evidence of underlying chronic *tropical phagedenic ulcer* whereas in cases from the Southern Sudan such ulcers were fairly frequent; this has also been the experience of Nabri (1968). The situation in the Southern Sudan in this respect is similar to that in neighbouring East Africa (Burkitt, 1966). Since most of the Sudanese work as farmers or herdsmen, they are subjected to repeated trauma, particularly on the legs, and as Hickey (1959) had noticed *scars* in these sites are common in Sudanese men this could well be an aetiological factor. Other possible predisposing factors were: (a) Burns: in 10 cases, squamous cell carcinomata were present in association with burn scars on the trunk. The way these burns were caused was interesting. They occurred in Southerners living in the jungle, where because of fear of wild animals they lit charcoal fires and slept around them; they developed the burns by overturning whilst asleep at night. Oettlé (1963) mentioned the development of squamous cell carcinomata in burn scars in South African Bantus who were epileptics and fell on to open fires on the ground or hut floors. (b) Xeroderma pigmentosa: 8 cases, 4 in children and 4 in adults, were encountered where this was associated with squamous cell carcinoma. This association has also been remarked upon by Oettlé (1963). (c) Albinism: 12 cases were seen, 2 in a brother and sister twins. One patient had, in addition to the squamous cell carcinoma, both basal cell carcinoma and malignant melanoma. The high susceptibility of albinos to skin cancer has also been reported by Oettlé (1963) in South African Bantus.

The frequency of squamous cell carcinoma of the conjunctiva was relatively high in our series and the vast majority of these cases came from the Northern Sudan. This is perhaps related to exposure to sunlight as the conjunctiva, being relatively unpigmented, is more susceptible to actinic rays. Another possible factor is the dry climate and the irritation to the eyes caused by the frequent sandstorms and dust in the atmosphere in this part of the country, a large part of which lies within the Sahara Desert. The possible aetiological relationship between climatic conditions and conjunctival carcinoma in the Northern Sudan has also been pointed out by Daoud and Osman (1970).

It was not surprising to find that the majority of penile cancers in our series occurred in patients from the Southern Sudan and this has also been the experience of Nabri (1968). In this part of the country, where the population is largely non-Muslim, circumcision is practiced infrequently. By contrast, in the predominantly Muslim Northern Sudan male circumcision is the rule. The protective role of circumcision against penile cancer has been adequately outlined in reports from East Africa (Dodge and Linsell, 1963; Dodge, Linsell and Davies, 1963), Egypt (Abul Nasr, 1961) and the Sudan (Lynch *et al.*, 1963). It has also been well demonstrated in Malaya, where the incidence of penile cancer in Muslim Malays (circumcised) was found to be extremely low while in the Chinese Malay (non-circumcised) it was fairly high (Marsden, 1958).

Almost all the basal cell carcinomata in our series were in patients from the Northern Sudan, particularly in those of a fair skin colour (Arab origin), this tumour being rare indeed in Southerners (Negroid origin). This is in keeping with the experience in East Africa, where Burkitt (1966) mentions that he has seen this tumour only once in an African during a period of 20 years. This may point to the involvement of a genetic factor or to the degree of pigmentation of the skin in relation to sunlight. As regards site, basal cell carcinomata in our series occurred predominantly in the region of the head and neck. This is similar to the situation elsewhere and would suggest that exposure to sunlight might be an aetiological agent.

The preponderance of malignant melanoma in the sole of the foot in our series, together with the fact that more than half the cases occurred in the two Provinces in the western part of the Sudan where people work almost entirely as wandering nomads and seasonal working farmers, point to the possibility of repeated trauma being a causative factor, particularly since many of those people walk and work barefooted. However, this factor—suggested by Hewer (1935)— cannot be the whole explanation as the same predilection for the sole is encountered in American Negroes, many of whom have sedentary jobs and most of whom wear shoes (Steiner, 1954). Lewis (1967) postulated that the important factor was the presence of pre-existing naevi in the soles of feet in the Africans. A combination of both factors might be at work. As regards melanoma of the face, its frequency in our series is lower than in the European but higher than in the Negroid African; this might be a reflection of several factors, including exposure to sunlight, degree of pigmentation and racial differences.

All our 36 cases of Kaposi's sarcoma occurred in males. This is similar to reports from other countries, both in Africa and elsewhere where male exclusiveness or preponderance as regards this tumour has been established (Taylor and Kyalwazi, 1972). Whether this situation is genetically determined or is an expression of occupational and environmental factors is uncertain. Only one of our cases was a child and although our figures are small for any generalization, it is interesting to note in this respect that Kaposi's sarcoma is relatively frequent in children in Uganda (Taylor and Kyalwazi, 1972). Most of the cases in our series occurred in patients from the Southern Sudan. This fits in with the zone in Tropical Africa, for instance Uganda (Lothe and Murray, 1962; Cook, 1966) and Congo (Thijs, 1957) where this tumour is known to be particularly prevalent. It was interesting to find that a number of patients from the Northern Sudan in whom this neoplasm was encountered, gave a history of having been to the South at some stage in their lives and that 2 of them have paid regular visits to the South as sailors. This would be in favour of an environmental or infective aetiological basis rather than of a genetic background.

Finally, it should be mentioned that the study of superficial cancer in the Sudan, by pointing to some differences in the distribution of certain tumours in the Northern and Southern regions of the country, regions which contrast both geographically and ethnologically, emphasizes the possible role played by racial and geographical factors in the genesis of cancer. In general, the pattern in the Southern Sudan seems to approximate more to that of the neighbouring African countries than to that of the Northern Sudan. On the other hand, the pattern in the Northern Sudan seems to be a mixture of the situation as it occurs in Caucasians and Negroids, though perhaps generally sharing more in common with the former. The Sudan, by virtue of its varied populace and geography, seems to be particularly suited for the study of the geographical pathology of malignant disease and it is hoped that more useful information will be forthcoming in that direction.

We are grateful to Mrs Beatrice Abdel Malik and Salah Adam for typing the manuscript.

### REFERENCES

Abul Nasr, A. L. (1961) Cancer of the Penis in Egypt. Kasr-El-Aini, *J. Surg.*, **2**, 271.

Burkitt, D. (1966) A Great Pathological Frontier. *Postgrad. med. J.*, **42**, 543.

Cook, J. (1966) Kaposi's Sarcoma, *J. R. Coll. Surg.*, **11**, 185.

Daoud, E. H., El Hassan, A. M., Zak, F. & Zakova, N. (1968) Aspects of Malignant Disease in the Sudan. In *Cancer in Africa*. Ed. P. Clifford *et al.* Nairobi: *E. Af. med. J.* & E. Afr. Publishing House.

Daoud, E. H. & Osman, A. A. (1970) Preliminary Report on Conjunctival Carcinoma in Northern Sudan. *Sudan med. J.*, **8**, 82.

Davies, J. N. P., Tank, R., Meyer, R. & Thurston, P. (1968) Cancer of the Integumentary Tissues in Uganda: The Basis for Prevention. *J. natn. Cancer Inst.*, **41**, 31.

Dodge, O. G. & Linsell, C. A. (1963) Carcinoma of the Penis in Uganda and Kenya Africans. *Cancer, N.Y.*, **16**, 1255.

Dodge, O. G., Linsell, C. A. & Davies, J. N. P. (1963) Circumcision and the Incidence of Carcinoma of the Penis and Cervix. *E. Afr. med. J.*, **40**, 444.

Dolbey, R. V. & Moore, A. W. (1924) The Incidence of Cancer in Egypt. *Lancet*, i, 587.

Harnett, W. L. (1952) *Survey of Cancer in London.* London: British Empire Cancer Campaign.

Hickey, B. B. (1959) Malignant Epithelial Tumours in the Sudanese. *Ann. R. Coll. Surg. Eng.*, **24**, 303.

Hewer, T. F. (1935) Malignant Melanoma in Coloured Races. The Role of Trauma in its Causation. *J. Path. Bact.*, **11**, 473.

Lewis, M. G. (1967) Malignant Melanoma of the Foot in Uganda. The Role of Pigmentation. *Br. J. Cancer*, **21**, 483.

Linsell, C. A. (1968) Cancer in Kenya. In *Cancer in Africa*. Ed. P. Clifford *et al.* Nairobi: *East Afr. med. J.* and East African Publishing House.

Lothe, F. & Murray, J. F. (1962) Kaposi's Sarcoma: Autopsy Findings in the African. *Acta Un. Int. Cancer*, **18**, 429.

Lynch, F. W., Seidman, H. & Hammound, E. C. (1970) Incidence of Cutaneous Cancer in Minnesota. *Cancer, N.Y.*, **25**, 83.

Lynch, J. B., El Hassan, A. M. & Omer, A. (1963) Cancer in the Sudan. *Sudan med. J.*, **2**, 29.

Malik, M. O. A. (1973) Death Certification in Khartoum Civil Hospital. *Sudan med. J.* In the press.

Marsden, A. T. H. (1958) The Geographical Pathology of Cancer in Malaya. *Br. J. Cancer*, **12**, 162.

Ministry of Health, Sudan (1970) Annual Report of Vital and Health Statistics Division, Khartoum.

Morris, G. C., Jr. & Horn, R. C. (1951) Malignant Melanoma in the Negro. Review of Literature and Report of 9 Cases. *Surgery*, **29**, 223.

Nabri, I. A. (1968) Pattern of Surgical Diseases in Two Separate Areas of the Sudan. A Study in Geographic Pathology. *Int. Path.*, **9**, 52.

Oettlé, A. G. (1963) Skin Cancer in Africa. *Natn. Cancer Inst. Monog.* No. 10, p. 197.

Oettlé, A. G. (1964) Cancer in Africa, especially in Regions South of the Sahara. *J. natn. Cancer Inst.*, **33**, 383.

OLUWASANAMI, J. C., WILLIAMS, A. O. & ALLI, A. F. (1969) Superficial Cancer in Nigeria. *Br. J. Cancer*, **23**, 714.

PACK, G. T., LENSON, N. & GERBER, D. M. (1952) Regional Distribution of Moles and Melanomas. *Archs Surg.*, **65**, 862.

PRATES, M. D. (1958) Malignant Neoplasms in Mozambique. *Br. J. Cancer*, **12**, 177.

SCHREIBER, M. M., SHAPIRE, S. I., BERRY, C. Z., DAHLEN, R. F. & FRIEDMAN, R. P. (1971) The Incidence of Skin Cancer in Southern Arizona (Tucson). *Archs Derm. Syph.*, **104**, 124.

SHAPIRO, M. P., KEEN, P., COHEN, L. & MURRAY, J. F. (1953) Skin Cancer in South African Bantus. *Br. J. Cancer*, **7**, 45.

STEINER, P. E. (1954) *Cancer, Race and Geography*. Baltimore: Williams and Wilkins Co.

TAYLOR, J. F. & KYALWAZI, S. K. (1972) Kaposi's Sarcoma. In *Medicine in a Tropical Environment*. Ed. A. G. Shaper *et al.* London: B.M.A.

THIJS, A. (1957) Kaposi's Angiosarcomatosis in the Belgian Congo and Ruanda-Urundi. *Ann. Soc. belge med.*, **37**, 295.

ZAKOVA, N., EL HASSAN, A. M., ZAK, F. & SATTI, A. (1968) Autopsy Findings in Khartoum. *Sudan med. J.*, **6**, 119.